# Supplementary material for: Accurate interpretation of within-host dissemination using barcoded bacteria
Source: mSystems. 2025 Dec 23;11(2):e01460-25. doi: 10.1128/msystems.01460-25 (PMC12911355; doi:10.1128/msystems.01460-25)
Supplement: Code S1 — Code for DREX score calculation. [file msystems.01460-25-s0001.rtf]

##Updated 11/10/25library(tibble)#set.seed() ##Variables. All are csv files#Column indexes where reference samples are located from InputTableFileNameWhereAreReferences <- 1:4##Barcode frequency count table (first column = barcode, all other columns = samples) that contains NO noise. Every barcode will be considered real. If you have run the STAMPR pipeline this is typically called FrequenciesWithoutNoiseNoiselessTableFileName  <- "FrequenciesWithoutNoise_Combined.csv"##Input to STAMPR pipeline. Contains some noise that is corrected out by Resiliency algorithm. This file is only used to define the referenceInputTableFileName <- "NoHopFreq_KPPR1_07292024.csv"##Same metadata file as used in GD calculation. Contains 4 columns. First is "Sample" = name of the sample, must match FrequenciesWithoutNoise column names. Second is "Group" = the mouse number. Third is "Order" = number for the tissue (e.g., all livers get the #1, all spleens get the #2, etc.). Fourth is "BioSample" which has all the additional metadata you might want to add. The "Group" variable controls what comparisons get made (only within the same group)MetaDataFileName <- "Metadata_KPPR1_Combined.csv"##Table containing founding population sizes. Must have the column named as "Ns"NsTableFileName <- "NsNb_Combined.csv"##Desired name of output fileAnswerFinalOutputFileName <- "Caity_AnswerFinal.csv"#Set to NULL, otherwise specify the sample name that all other samples will be compared to. This was used for the simulations.AllToOne <- NULL##Set to TRUE if you want to ensure that the number of barcodes in the sample is not substantially greater than would be expected for the given FP value. This is essential if you are using Nb instead of Ns, but otherwise set to FALSEAdjustForNsLogic <- FALSE###Combine Reference VectorsInputCountTable <- read.csv(InputTableFileName, row.names = 1)ReferenceVector <- InputCountTable[,WhereAreReferences]if(class(ReferenceVector) != "numeric") {ReferenceVector <- rowSums(ReferenceVector) / sum(rowSums(ReferenceVector)) }##Define Genetic Distance MetricGeneticDistance <-function(f1, f2) {  f1 <- f1/sum(f1)  f2 <- f2/sum(f2)  cosTheta=sum(sqrt(f1*f2))  if(1-cosTheta < 0) {cosTheta <- 1}  #chorddistance=2*sqrt(2)/pi*sqrt(1-cosTheta)  ##Modified so range is from 0 to 1  chorddistance=sqrt(1-cosTheta)  chorddistance}##Read MetaData, Noiseless table, and Ns tableMetaData <- read.csv(MetaDataFileName)NoiselessCountTable <- read.csv(NoiselessTableFileName)NsTable <- read.csv(NsTableFileName, row.names = 1)##Create PairsSampleNamesOrdered <- MetaData$Sample[order(MetaData$Group, MetaData$Order)]TableOfComparisons <- data.frame(vector1 = rep(SampleNamesOrdered,length(SampleNamesOrdered)), vector2 = rep(SampleNamesOrdered, each = length(SampleNamesOrdered)))FilterToGroups <- function(vec) {  group1 <- MetaData$Group[which(MetaData$Sample == as.character(vec[1]))]  group2 <- MetaData$Group[which(MetaData$Sample == as.character(vec[2]))]  c(group1, group2)}GroupTable <- t(apply(TableOfComparisons,1, FilterToGroups))FilteredComparisons <- TableOfComparisons[GroupTable[,1] == GroupTable[,2],]FilteredComparisons <- FilteredComparisons[FilteredComparisons$vector1 != FilteredComparisons$vector2,]if(!is.null(AllToOne)) {    FilteredComparisons <- FilteredComparisons[which(FilteredComparisons$vector1 == AllToOne),]    }CalculateZForOnePair <- function(PairName){print(PairName)Sample1Name <- as.character(PairName[1])Sample2Name <- as.character(PairName[2])  Vec1 <- NoiselessCountTable[,which(colnames(NoiselessCountTable)==Sample1Name)]Vec2 <- NoiselessCountTable[,which(colnames(NoiselessCountTable)==Sample2Name)]GD <- GeneticDistance(Vec1, Vec2)NsVec1 <- round(NsTable[which(rownames(NsTable) == Sample1Name),]$Ns)NsVec2 <- round(NsTable[which(rownames(NsTable) == Sample2Name),]$Ns)if(AdjustForNsLogic) {###Ensuring that Ns values make sense. This step performs simulations with the given value of Ns to determine the number of unique barcodes (opposite of how Ns is calculated). It then adjusts the output vectors such that only this many barcodes are present (taking the top barcodes). If you actually use the Ns value determine by getFP, this should have very minimal effect. However, if you are tricking the computer by using Nb instead, or for whatever reason have weird Ns values, this step ensures that your real output vectors have the same number of barcodes as the simulated ones. `%NotIn%` <- Negate(`%in%`)Vec1Sim <- rmultinom(50, NsVec1, ReferenceVector)Vec1Sim[Vec1Sim != 0] <- 1NBarcodesVec1Sim <- round(mean(colSums(Vec1Sim)))LowestVec1_TF <- 1:length(Vec1) %NotIn% order(Vec1, decreasing = TRUE)[1:NBarcodesVec1Sim]Vec1[LowestVec1_TF] <- 0  Vec2Sim <- rmultinom(50, NsVec2, ReferenceVector)Vec2Sim[Vec2Sim != 0] <- 1NBarcodesVec2Sim <- round(mean(colSums(Vec2Sim)))LowestVec2_TF <- 1:length(Vec2) %NotIn% order(Vec2, decreasing = TRUE)[1:NBarcodesVec2Sim]Vec2[LowestVec2_TF] <- 0  }###GD equalized is the GD that is calculated AFTER barcodes have been removed in the "AdjustForNsLogic" step. You can always do a sanity check to make sure that GD_Equalized > GD (if this was set to TRUE, otherwise they will be the same number)GD_Equalized <- GeneticDistance(Vec1, Vec2)###Simulate samples with eqivalent founding populationsSimulateNullGDs <- function() {  Vec1Sim <- as.numeric(rmultinom(1, NsVec1, ReferenceVector))  Vec2Sim <- as.numeric(rmultinom(1, NsVec2, ReferenceVector))  SimGD <- GeneticDistance(Vec1Sim, Vec2Sim)    Vec1SimPresenceAbsence <- as.numeric(Vec1Sim != 0)  Vec2SimPresenceAbsence <- as.numeric(Vec2Sim != 0)    SharedBarcodesLogicVectorSim <- (Vec1SimPresenceAbsence & Vec2SimPresenceAbsence) == 1  WeightedFractionSharedVec1Sim <- sum(Vec1Sim[SharedBarcodesLogicVectorSim]) / sum(Vec1Sim)  WeightedFractionSharedVec2Sim <- sum(Vec2Sim[SharedBarcodesLogicVectorSim]) / sum(Vec2Sim)    UnweightedFractionSharedVec1Sim <- sum(Vec1SimPresenceAbsence[SharedBarcodesLogicVectorSim]) / sum(Vec1SimPresenceAbsence)  UnweightedFractionSharedVec2Sim <- sum(Vec2SimPresenceAbsence[SharedBarcodesLogicVectorSim]) / sum(Vec2SimPresenceAbsence)   c(SimGD, sum(SharedBarcodesLogicVectorSim), WeightedFractionSharedVec1Sim, WeightedFractionSharedVec2Sim, UnweightedFractionSharedVec1Sim, UnweightedFractionSharedVec2Sim)}##Change the number here if you want to change the number of simulationsSimGDVector <- data.frame(t(replicate(50, SimulateNullGDs())))colnames(SimGDVector) <- c("SimGD", "NumSharedSim", "WeightedFracSharedVec1Sim", "WeightedFracSharedVec2Sim", "UnweightedFracSharedVec1Sim", "UnweightedFracSharedVec2Sim" )Vec1PresenceAbsence <- as.numeric(Vec1 != 0)Vec2PresenceAbsence <- as.numeric(Vec2 != 0)SharedBarcodesLogicVector <- (Vec1PresenceAbsence & Vec2PresenceAbsence) == 1WeightedFractionSharedVec1 <- sum(Vec1[SharedBarcodesLogicVector]) / sum(Vec1)WeightedFractionSharedVec2 <- sum(Vec2[SharedBarcodesLogicVector]) / sum(Vec2)UnweightedFractionSharedVec1 <- sum(Vec1PresenceAbsence[SharedBarcodesLogicVector]) / sum(Vec1PresenceAbsence)UnweightedFractionSharedVec2 <- sum(Vec2PresenceAbsence[SharedBarcodesLogicVector]) / sum(Vec2PresenceAbsence)z_GD <- (GD_Equalized - mean(SimGDVector$SimGD)) / sd(SimGDVector$SimGD)z_NumShared <- (sum(SharedBarcodesLogicVector) - mean(SimGDVector$NumSharedSim)) / sd(SimGDVector$NumSharedSim)z_WFS_Vec1 <- (WeightedFractionSharedVec1 - mean(SimGDVector$WeightedFracSharedVec1Sim)) / sd(SimGDVector$WeightedFracSharedVec1Sim)z_WFS_Vec2 <- (WeightedFractionSharedVec2 - mean(SimGDVector$WeightedFracSharedVec2Sim)) / sd(SimGDVector$WeightedFracSharedVec2Sim)z_UFS_Vec1 <- (UnweightedFractionSharedVec1 - mean(SimGDVector$UnweightedFracSharedVec1Sim)) / sd(SimGDVector$UnweightedFracSharedVec1Sim)z_UFS_Vec2 <- (UnweightedFractionSharedVec2 - mean(SimGDVector$UnweightedFracSharedVec2Sim)) / sd(SimGDVector$UnweightedFracSharedVec2Sim)c(NsVec1, NsVec2,   GD, GD_Equalized, mean(SimGDVector$SimGD), z_GD,  sum(SharedBarcodesLogicVector),  mean(SimGDVector$NumSharedSim), z_NumShared,  WeightedFractionSharedVec1, mean(SimGDVector$WeightedFracSharedVec1Sim), z_WFS_Vec1,  WeightedFractionSharedVec2,  mean(SimGDVector$WeightedFracSharedVec2Sim), z_WFS_Vec2,  UnweightedFractionSharedVec1, mean(SimGDVector$UnweightedFracSharedVec1Sim), z_UFS_Vec1,  UnweightedFractionSharedVec2, mean(SimGDVector$UnweightedFracSharedVec2Sim), z_UFS_Vec2  )}Answer <- apply(FilteredComparisons, 1, CalculateZForOnePair)Answer <- t(Answer)colnames(Answer) <- c("NsVec1", "NsVec2",                       "GD","GD_Equalized", "SimGD", "z_GD",                       "NumBarcodesShared", "SimNumBarcodesShared", "z_NumBarcodesShared",                      "WFS_Vec1", "WFS_SimVec1", "z_WFS_Vec1",                       "WFS_Vec2", "WFS_SimVec2", "z_WFS_Vec2",                       "UFS_Vec1", "UFS_SimVec1", "z_UFS_Vec1",                      "UFS_Vec2", "UFS_SimVec2", "z_UFS_Vec2"                      )GetBioSample <- function(SampleName) {  MetaData$BioSample[which(MetaData$Sample == SampleName)]}Vector1Name <- sapply(FilteredComparisons$vector1, GetBioSample)Vector2Name <- sapply(FilteredComparisons$vector2, GetBioSample)AnswerFinal <- data.frame("Vector1" = FilteredComparisons$vector1, "Vector2" = FilteredComparisons$vector2, Vector1Name, Vector2Name, Answer)OrdersVec1 <- MetaData$Order[match(AnswerFinal$Vector1, MetaData$Sample)]OrdersVec2 <- MetaData$Order[match(AnswerFinal$Vector2, MetaData$Sample)]GroupVec1 <- MetaData$Group[match(AnswerFinal$Vector1, MetaData$Sample)]GroupVec2 <- MetaData$Group[match(AnswerFinal$Vector2, MetaData$Sample)]Genotype <- MetaData$Genotype[match(AnswerFinal$Vector2, MetaData$Sample)]###Appends a "1" if a specific character (defined as KeyPattern) is found in any sampleKeyPattern <- "L"Highlight1 <- grep(KeyPattern, AnswerFinal$Vector1Name)Highlight2 <- grep(KeyPattern, AnswerFinal$Vector2Name)CombinedHighlight <- unique(c(Highlight1, Highlight2))HighlightVector <- rep(0, length.out = dim(AnswerFinal)[1])HighlightVector[CombinedHighlight] <- 1AnswerFinal <- data.frame(AnswerFinal, OrdersVec1, OrdersVec2, GroupVec1, GroupVec2, Genotype, HighlightVector)##Add CFUs and abscess if needed# # CFUVec1 <- log10(MetaData$CFU[match(AnswerFinal$Vector1, MetaData$Sample)])# CFUVec2 <- log10(MetaData$CFU[match(AnswerFinal$Vector2, MetaData$Sample)])# # AbscessVec1 <- MetaData$Abscess[match(AnswerFinal$Vector1, MetaData$Sample)]# AbscessVec2 <- MetaData$Abscess[match(AnswerFinal$Vector2, MetaData$Sample)]# # # AnswerFinal <- data.frame(AnswerFinal, CFUVec1, CFUVec2, AbscessVec1, AbscessVec2)###Create interactive plotwrite.csv(AnswerFinal, AnswerFinalOutputFileName, row.names = FALSE)##AnswerFinal is the output file. DREX scores as defined in the paper are in the "z_GD" column. There are other metrics spit out too. These are:#"NBarcodesShared" = number of barcodes shared between samples#"WFS" = "weighted fraction shared". This is basically the relative abundance in the sample of the NBarcodesShared. There are two WFS values. One for Vec1 and one for Vec2##UFS = "unweighted fraction shared". This is NBarcodesShared relative to the total number of barcodes in the sample. NOT considering their relative abundance. Like with WFS, here are 2 UFS values#NBarcodesShared, WFS, and UFS, also have a similar z-score confidence calculation in the respective columns (e.g., "z_WFS"). These aren't really talked about in the paper but can be nice to see. They are effectively the identical logic as DREX scores but instead of doing it for GD it is being calculated for the other dissemination variables.###END###Some old code for plotting purposes. library(ggplot2)library(plotly)library(RColorBrewer)library(viridis)Create4DPlot <- function(KeywordSubset, Xvals, Yvals) {  if (is.null(KeywordSubset)) {AnswerFinalSubset <- AnswerFinal} else{AnswerFinalSubset <- AnswerFinal[grep(KeywordSubset, AnswerFinal$Vector1Name),]}  test <- AnswerFinalSubset %>%                 mutate (text = paste("Vector1Name: ", Vector1Name,                        "\nVector2Name: ", Vector2Name,                       "\nGD: ", GD,                       "\nz_GD: ", z_GD,                       "\nCFUVec1: ", CFUVec1,                       sep="")) %>%           ggplot(aes(x = .data[[Xvals]],                     y = .data[[Yvals]],                text = text)) +        geom_point(aes(fill = CFUVec1, size = abs(z_GD)), shape = 21, stroke = .4) +        theme_bw()+        scale_fill_viridis(begin = min(AnswerFinalSubset$GD), end = max(AnswerFinalSubset$GD))ggplotly(test, tooltip = "text")}Create4DPlot("L", "z_GD", "GD")Create2DPlot <- function(KeywordSubset, Xvals, Yvals) {    if (is.null(KeywordSubset)) {AnswerFinalSubset <- AnswerFinal} else  {AnswerFinalSubset <- AnswerFinal[grep(KeywordSubset, AnswerFinal$Vector1Name),]}      test <- AnswerFinalSubset %>%         mutate (text = paste("Vector1Name ", Vector1Name,                          "\nVector2Name: ", Vector2Name,                         "\nGD: ", GD,                         "\nz_GD: ", z_GD,                         sep="")) %>%        ggplot(aes(x = .data[[Xvals]],                y = .data[[Yvals]],               text = text)) +    geom_point(aes(fill = as.factor(Genotype)), size = 3, alpha = .7) +    theme_bw()    #scale_fill_viridis(begin = min(AnswerFinalSubset$GD), end = max(AnswerFinalSubset$GD))      ggplotly(test, tooltip = "text")  }Create2DPlot(NULL, "z_GD", "GD")####Boxplots for abscessesUniqueVector1 <- unique(AnswerFinal$Vector1)#AnswerFinalSubset <- AnswerFinal[match(UniqueVector1, AnswerFinal$Vector1),]AnswerFinalSubset <- AnswerFinal[grep(KeywordSubset, AnswerFinal$Vector1Name),]test <- AnswerFinalSubset %>%        mutate(zGDPass = (z_GD < -5)) %>%          ggplot(aes(x = Genotype, y = z_GD)) +          geom_boxplot() +              geom_point(aes(color = as.factor(zGDPass)))+           theme_bw()test####plotting for simulationsplot(x = AnswerFinal$NsVec2, y = AnswerFinal$z_GD)hist(AnswerFinal$z_GD)###Calculate mean of each stat for every unique pair    OrdersVec1 <- MetaData$Order[match(AnswerFinal$Vector1, MetaData$Sample)]  OrdersVec2 <- MetaData$Order[match(AnswerFinal$Vector2, MetaData$Sample)]  CombinedOrders <- (paste(OrdersVec1, OrdersVec2, sep = ":"))    GetMeanOfOrders <- function(OrderPair) {        mean_GD <- mean(AnswerFinal$GD[which(CombinedOrders == OrderPair)])    mean_z_GD <- mean(AnswerFinal$z_GD[which(CombinedOrders == OrderPair)])    mean_NBS <- mean(AnswerFinal$NumBarcodesShared[which(CombinedOrders == OrderPair)])    mean_z_NBS <- mean(AnswerFinal$z_NumBarcodesShared[which(CombinedOrders == OrderPair)])        mean_WFS_Vec1 <- mean(AnswerFinal$WFS_Vec1[which(CombinedOrders == OrderPair)])    mean_z_WFS_Vec1 <- mean(AnswerFinal$z_WFS_Vec1[which(CombinedOrders == OrderPair)])        mean_WFS_Vec2 <- mean(AnswerFinal$WFS_Vec2[which(CombinedOrders == OrderPair)])    mean_z_WFS_Vec2 <- mean(AnswerFinal$z_WFS_Vec2[which(CombinedOrders == OrderPair)])        mean_UFS_Vec1 <- mean(AnswerFinal$UFS_Vec1[which(CombinedOrders == OrderPair)])    mean_z_UFS_Vec1 <- mean(AnswerFinal$z_UFS_Vec1[which(CombinedOrders == OrderPair)])        mean_UFS_Vec2 <- mean(AnswerFinal$UFS_Vec2[which(CombinedOrders == OrderPair)])    mean_z_UFS_Vec2 <- mean(AnswerFinal$z_UFS_Vec2[which(CombinedOrders == OrderPair)])        c(mean_GD, mean_z_GD, mean_NBS, mean_z_NBS, mean_WFS_Vec1, mean_z_WFS_Vec1, mean_WFS_Vec2, mean_z_WFS_Vec2, mean_UFS_Vec1, mean_z_UFS_Vec1, mean_UFS_Vec2, mean_z_UFS_Vec2)      }   MeanAnswerTable <- rownames_to_column(data.frame(t(sapply(unique(CombinedOrders), GetMeanOfOrders))))    colnames(MeanAnswerTable) <- c("OrderPair", "mean_GD", "mean_z_GD", "mean_NBS", "mean_z_NBS", "mean_WFS_Vec1", "mean_z_WFS_Vec1", "mean_WFS_Vec2", "mean_z_WFS_Vec2", "mean_UFS_Vec1", "mean_z_UFS_Vec1", "mean_UFS_Vec2", "mean_z_UFS_Vec2")  
