# Supplementary material for: Accurate interpretation of within-host dissemination using barcoded bacteria
Source: mSystems. 2025 Dec 23;11(2):e01460-25. doi: 10.1128/msystems.01460-25 (PMC12911355; doi:10.1128/msystems.01460-25)
Supplement: Supplemental Figures — Figures S1 and S2. [file msystems.01460-25-s0002.docx]

**Supplemental material**

**Supplementary Figures and Legends**

| **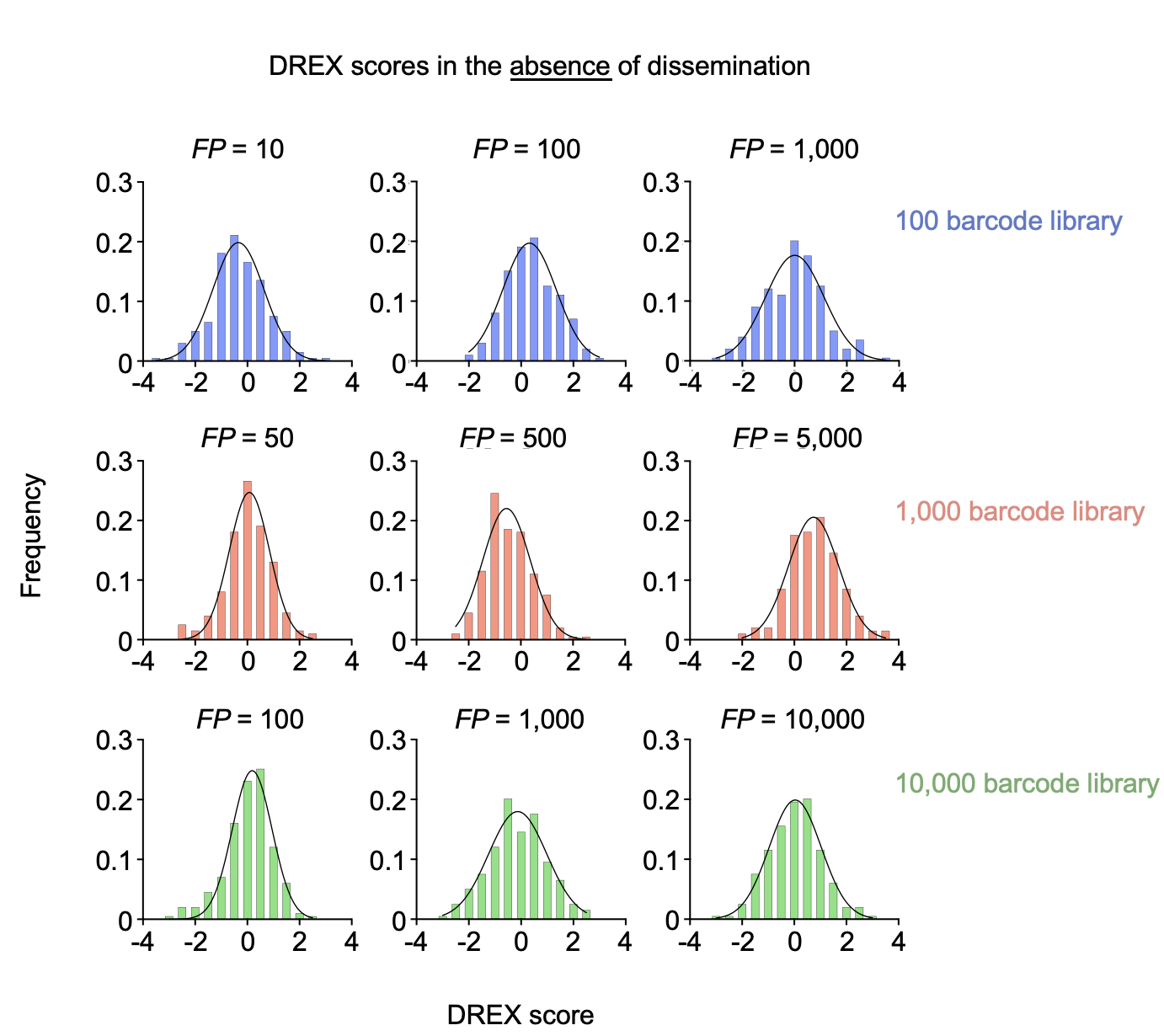** |
| --- |
| **Figure S1. Additional DREX scores for simulated samples without dissemination.** Simulated samples across various founding population sizes and library diversities were generated and DREX scores were calculated (200 simulations per graph). Based on these data, a DREX score less than -4 was considered as a threshold where sharing of barcodes is greater than expected by random chance. A DREX score greater than 4 was considered as the threshold where sharing of barcodes is less than expected by random chance |

| 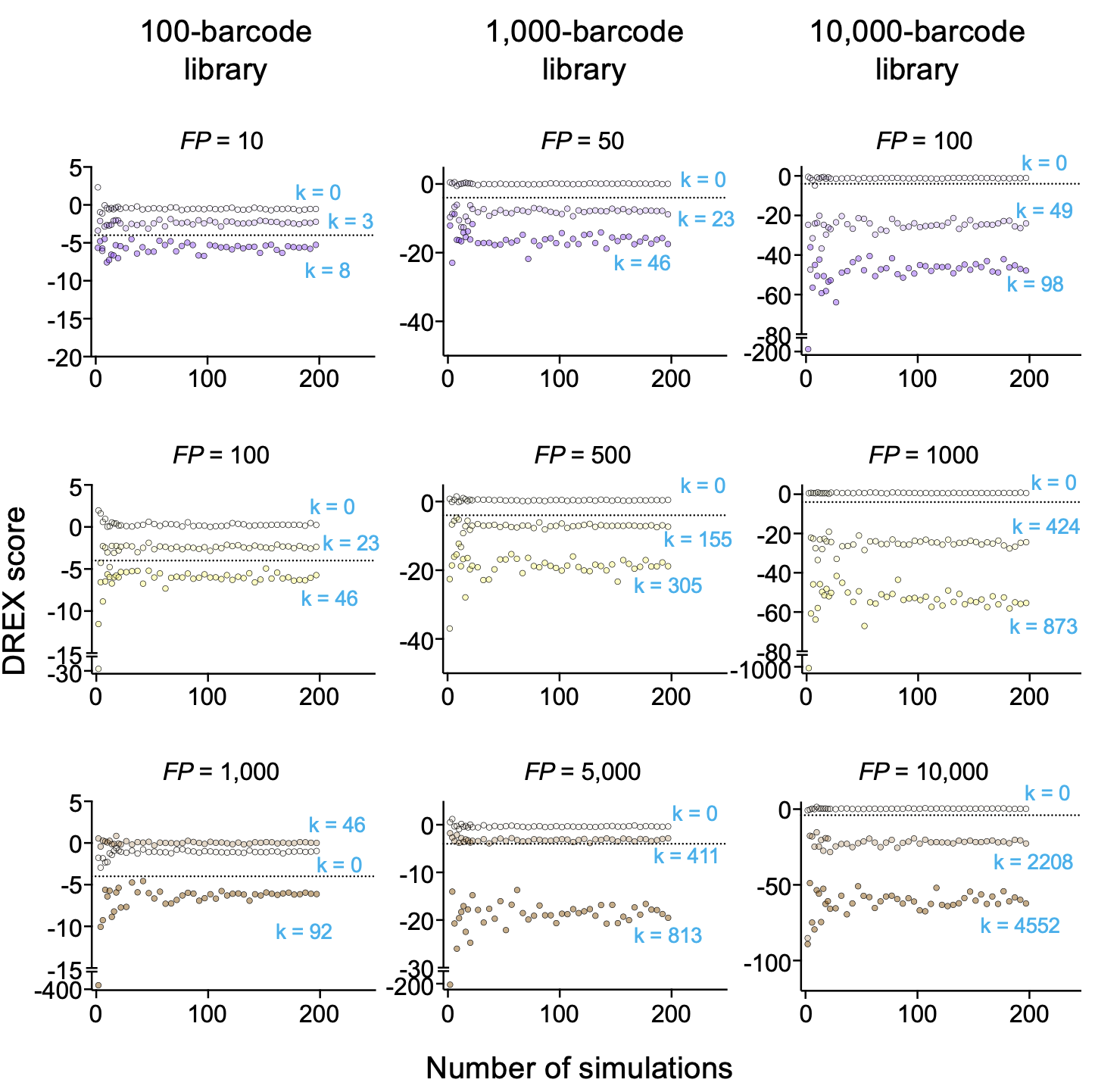 |
| --- |
| **Figure S2. Using different numbers of simulations for DREX score calculation**  Using the simulated dataset from Figure 4, we varied the number of simulations that are used for DREX score calculation from 2 to 200. The value of *k* represents the number of barcodes that were transferred (i.e., increasing numbers of disseminated clones). In this study, we used 50 simulations for DREX score calculation, which is large enough to provide a reliable DREX score while minimizing computational time. |

**Supplementary Code 1. Code for DREX score calculation**
